# Transsacral Bar Fixation for Osteoporotic H-Type Sacral Fractures: A Viable Alternative to Spinopelvic Fixation

**DOI:** 10.3390/jcm14186503

**Published:** 2025-09-16

**Authors:** Martin Naisan, Felix Schmitz, Yazan Noufal, Yama Afghanyar, Matthias Fröhlich, Marcus Richter, Philipp Drees, Philipp Hartung

**Affiliations:** 1 Spine Center, St. Josefs Hospital, 65189 Wiesbaden, Germany; ynoufal@joho.de (Y.N.); mrichter@joho.de (M.R.); phartung@joho.de (P.H.); 2 Department of Orthopedics, St. Josefs Hospital, 65189 Wiesbaden, Germany; fschmitz@joho.de; 3 Center of Orthopedics and Traumatology, University Medical Center Mainz, 55116 Mainz, Germany; yama.afghanyar@unimedizin-mainz.de (Y.A.); philipp.drees@unimedizin-mainz.de (P.D.); 4 Department of Trauma and Orthopedic Surgery, Cologne-Merheim Medical Centre (CMMC), University of Witten/Herdecke, 51109 Cologne, Germany; froehlichm@kliniken-koeln.de

**Keywords:** sacral fractures, fragility fractures, osteoporosis, transsacral bar, spinopelvic fixation, elderly, computer navigation

## Abstract

**Background**: Fragility fractures of the pelvis (FFP) are an increasing challenge in aging societies. Among these, FFP type 4B (“H-shaped” sacral fractures) represent the most unstable subtype, characterized by bilateral sacral ala fractures with transverse dissociation. Optimal fixation strategies remain debated, as spinopelvic fixation provides maximal stability but is invasive, while iliosacral screws often fail in osteoporotic bone. Trans-sacral bar (TSB) fixation has been proposed as a less invasive alternative, though evidence for its use in FFP 4B remains limited. **Methods**: We conducted a retrospective single-center study of 31 elderly patients (mean age 77.9 years; 87.1% female) with CT-confirmed FFP type 4B fractures treated between 2015 and 2022 using navigation-guided TSB constructs. Surgical configurations included hybrid fixation (TSB + bilateral iliosacral screws, *n* = 25) and dual-bar fixation (*n* = 6). Outcomes included perioperative complications, implant survival, radiographic healing, pain, and mobility at 3 and 12 months. Opportunistic CT-derived Hounsfield units (HU) were used to assess bone quality. **Results**: All patients had severe osteoporosis (mean HU 75.8 ± 30.1). Mean operative time was 71 min, and mean hospitalization was 9.1 days. No intraoperative or postoperative complications occurred, and no implant loosening, migration, or revision surgeries were required. At 3 months, mean pain score was 1.9, further decreasing to 1.1 at 12 months; 60.9% of patients reported complete pain resolution. Mobility improved in most cases, with 80.6% discharged with a walker or crutches. Radiographic follow-up confirmed stable healing in all patients. **Conclusions**: Navigation-guided TSB-based fixation provided stable osteosynthesis with excellent implant survival, significant pain relief, and early mobilization in elderly patients with FFP type 4B fractures. Hybrid and dual-bar constructs both achieved reliable outcomes. TSB fixation thus represents a safe and effective alternative to spinopelvic fixation in this fragile population. Larger multicenter prospective studies are warranted to confirm these findings and refine fixation strategies.

## 1. Introduction

Fragility fractures of the pelvis (FFP) have emerged as a major clinical and socioeconomic challenge in aging societies. With steadily increasing life expectancy worldwide, the incidence of osteoporotic pelvic fractures is rising sharply. These fractures are recognized not only as an orthopedic condition but also as a major public health issue due to their far-reaching implications for healthcare systems, social care, and long-term patient outcomes.

Epidemiological studies from Europe illustrate this trend: a nationwide German analysis found a substantial rise in pelvic fracture rates between 2002 and 2018, particularly in patients aged over 80 years [1]. Similarly, Finnish registry data covering more than 33,000 adults documented a significant increase in pelvic fractures over a 17-year period [2], while Swedish population data demonstrated diverging trends with pelvic and femur fractures showing increasing incidence among the elderly [3]. These studies collectively highlight how pelvic fragility fractures have become a frequent and debilitating condition, now affecting up to 40 per 100,000 persons annually in populations over the age of 65 [4].

In addition to Europe, comparable increases have been observed globally, reflecting the worldwide demographic shift toward older populations. Countries in Asia and North America report similar trends, with projections suggesting that the burden of osteoporotic pelvic fractures will double in the coming decades as the proportion of people aged >75 years grows dramatically. This epidemiological development is paralleled by an increasing demand for hospital resources, prolonged inpatient stays, and escalating healthcare costs. Compared to hip fractures, pelvic fractures remain less extensively studied, yet their clinical impact and long-term consequences appear to be equally profound.

The clinical relevance of FFP is considerable. They are often precipitated by low-energy trauma, such as a simple fall from standing height, or even occur atraumatically in the context of severe osteoporosis [5]. In some cases, patients present with vague pain and reduced mobility without a distinct traumatic event, complicating timely diagnosis. The resulting pain and immobility can trigger a cascade of complications including pneumonia, venous thromboembolism, urinary tract infections, and pressure ulcers [6]. Functional decline, loss of independence, and transition to long-term institutional care are common consequences, especially in the frail elderly population.

Mortality rates after pelvic fragility fractures are comparable to those observed after hip fractures, with recent systematic reviews reporting one-year mortality rates approaching 10–15% in this population [7]. The burden is not restricted to mortality alone; reduced quality of life, loss of autonomy, and chronic pain are frequent sequelae. In many cases, patients never return to their pre-injury level of activity, and a considerable proportion require permanent walking aids or transfer to nursing homes. This highlights that pelvic fragility fractures, although traditionally underestimated compared to hip fractures, represent a devastating injury with implications extending well beyond the acute hospitalization.

Among posterior pelvic ring injuries, bilateral lesions are particularly destabilizing. The FFP classification system, first proposed by Rommens and Hofmann and recently updated [8,9], stratifies pelvic fragility fractures into four main categories. Within type 4, the 4B subtype (“H-shaped” fracture) is characterized by bilateral vertical sacral ala fractures connected by a transverse component through the sacral body. This unique morphology results in a structural dissociation of the spinal column from the pelvic ring, analogous to high-energy spinopelvic dissociation [10].

This morphology is not only mechanically unstable but also radiographically challenging to identify. Because fracture lines can be subtle on plain radiographs, cross-sectional imaging with CT or MRI is frequently required to establish the diagnosis [11]. Missed or delayed recognition of these fractures can lead to secondary displacement, aggravating the instability and clinical consequences.

Clinically, H-shaped sacral fractures are associated with severe instability, intractable pain, and profound limitations in mobility. Left untreated, patients are at high risk of progressive deformity [12], chronic pain, and complications associated with prolonged recumbency [6]. These features explain why FFP 4B fractures are almost universally considered for surgical stabilization rather than conservative management [13].

Given their severity, H-shaped fractures are often viewed as the “osteoporotic counterpart” of traumatic spinopelvic dissociation seen in younger individuals after high-energy injuries. However, the osteoporotic bone quality in elderly patients introduces additional complexity, limiting the reliability of conventional fixation strategies that were originally designed for younger trauma populations.

Osteoporosis is the dominant predisposing factor for FFP. Age-related bone loss, combined with sarcopenia, frailty, and comorbidities such as diabetes and cardiovascular disease, substantially increases fracture risk [14,15]. Additional contributors include reduced balance, polypharmacy with sedatives, and neuromuscular decline—all of which increase the risk of falls in elderly individuals. Unlike high-energy pelvic trauma, fragility fractures occur under minimal force, often from simple daily activities.

Large-scale epidemiological studies confirm the high prevalence of low bone mineral density (BMD) worldwide, with estimates suggesting that over 20% of women above 65 years suffer from osteoporosis [16,17]. In men, osteoporosis is less frequently diagnosed but still clinically significant, particularly because fracture-related mortality is higher in male patients. The global burden of osteoporosis is projected to expand sharply, with the absolute number of individuals at risk of fragility fractures expected to increase in parallel with the aging population.

Histological and imaging studies have demonstrated that sacral bone mass is heterogeneously distributed, with relatively low trabecular density in the sacral ala, predisposing this region to insufficiency fractures [18,19]. This anatomical vulnerability helps to explain the characteristic patterns of sacral fragility fractures, where vertical ala fractures dominate.

Traditional dual-energy X-ray absorptiometry (DXA) remains the reference for diagnosing osteoporosis. However, DXA was not consistently available in many retrospective cohorts. Recent work shows that opportunistic CT-derived Hounsfield unit (HU) measurements provide a reliable surrogate marker of BMD, with thresholds ≤ 110 HU considered highly specific and values around 160–170 HU considered sensitive for osteoporosis detection [20]. This approach has become especially relevant for elderly fracture patients, since pelvic or lumbar CT imaging is routinely obtained during trauma assessment. Moreover, it allows retrospective analysis of bone density in clinical studies where DXA scans were unavailable, providing important insights into the pathophysiological role of osteoporosis in fragility fracture patterns.

The optimal stabilization method for H-shaped sacral fragility fractures remains debated. The main surgical options include:

- Iliosacral screws (IS): Minimally invasive and widely used, but biomechanical studies show that screw purchase is often insufficient in severely osteoporotic bone, leading to loosening or failure [10,21]. Cement augmentation may improve fixation strength but carries risks such as cement leakage and thermal injury [6].
- Trans-sacral bar (TSB) fixation: A bar spanning both sacroiliac joints achieves bicortical anchorage and distributes axial and torsional loads across the sacrum. This configuration is particularly attractive in osteoporotic bone, where the purchase of iliosacral screws alone is compromised [9,22]. Variants include single-bar, dual-bar, or hybrid constructs combining a TSB with bilateral iliosacral screws.
- Spinopelvic fixation (SPF): Lumbopelvic constructs provide maximal stability and are considered the traditional reference treatment in young patients with high-energy traumatic spinopelvic dissociations [23,24]. However, in elderly, frail patients, SPF carries considerable drawbacks—including longer operative times, increased blood loss, higher wound complication rates, and difficulties with postoperative mobilization [25,26].

Each of these techniques has its proponents and limitations. While iliosacral screws represent the least invasive option, their biomechanical reliability in osteoporotic bone is questionable. Spinopelvic fixation provides maximal stability but at the cost of invasiveness and perioperative morbidity. The trans-sacral bar technique, therefore, has attracted increasing attention as a compromise between stability and invasiveness, though evidence remains limited, especially for the most unstable FFP type 4B.

While recent series have demonstrated encouraging results with trans-sacral fixation in pelvic fragility fractures [9,22], most included heterogeneous patterns (FFP types 2–4). Data specifically focused on FFP type 4B (true H-fractures) remain scarce. Andresen et al. highlighted that bilateral trans-sacral screw fixation could achieve outcomes comparable to SPF with fewer complications, yet their series did not isolate H-fractures [13].

Biomechanical research emphasizes the importance of construct configuration. Single-bar constructs may be biomechanically insufficient in rotational stability [18], whereas two-point fixation (TSB + screws or dual bars) provides improved load sharing and resistance to torsional forces [24]. Clinical confirmation of these findings in the most unstable fragility fracture type is, however, lacking.

Thus, despite growing awareness of the clinical relevance of pelvic fragility fractures, robust evidence for optimal management of H-shaped sacral fractures remains absent. This gap underscores the necessity of focused clinical research specifically addressing FFP type 4B.

## 2. Materials and Methods

### 2.1. Study Design and Ethical Considerations

This was a single-center retrospective cohort analysis conducted at a tertiary academic spine and trauma center. The study was approved by the Ethics Committee of the Medical Association of Hessen (Reference Number: 2022-2899-zvBO, 31 March 2022) and conducted in accordance with the Declaration of Helsinki and Good Clinical Practice (GCP) guidelines. All participants provided written informed consent for treatment and use of anonymized data for research purposes.

### 2.2. Patient Selection

Patients ≥ 65 years with CT-confirmed osteoporotic H-type sacral fractures (FFP type 4B) treated between January 2015 and January 2022 with navigated trans-sacral bar (TSB) fixation were eligible. Osteoporotic etiology was confirmed by clinical history, imaging findings, and, where available, bone mineral density testing.

Exclusion criteria included:

- High-energy trauma mechanism;
- Pathological fractures due to metastatic disease, infection, or prior radiation therapy;
- Prior pelvic fixation;
- Incomplete imaging data or insufficient follow-up.

Fracture Classification and Surgical Indications

Fractures were classified using both the Fragility Fractures of the Pelvis (FFP) classification system [27], which emphasizes biomechanical and clinical factors, and the Osteoporotic Fractures (OF) Pelvis classification [28].

Fragility Fractures of the Pelvis (FFP) Classification:

The Fragility Fractures of the Pelvis (FFP) classification, introduced by Rommens and Hofmann in 2013, is a morphology-based system specifically designed for low-energy pelvic fractures in elderly patients with osteoporosis. It categorizes injuries into four main types of increasing mechanical instability:

- FFP Type I—Isolated anterior pelvic ring fracture;
- FFP Type II—Non-displaced posterior ring fracture;
- FFP Type III—Unilateral displaced posterior ring fracture;
- FFP Type IV—Bilateral posterior ring involvement.

Within type IV, the 4B subtype (“H-shaped” fracture) is characterized by bilateral vertical sacral fractures connected by a transverse component through the sacral body.

In this study, all patients presented with FFP type 4B fractures, the most unstable pattern, and therefore required surgical fixation.

Osteoporotic Fractures (OF) Pelvis Classification:

The OF Pelvis classification is a novel, treatment-oriented scoring system designed to guide management of osteoporotic pelvic fractures. It extends the principles of the established OF spine classification to the pelvic ring and integrates fracture morphology with patient-related parameters. Grades range from OF 1 (minor injury without instability) to OF 5 (complete spinopelvic dissociation).

In addition to morphology, the OF Pelvis Score incorporates pain severity, neurological deficits, baseline mobility, comorbidities, and frailty to produce a cumulative score. In this study, surgical fixation was performed in all patients with FFP type 4B fractures, corresponding to OF Grade 4 or higher, and supported by an OF Pelvis score exceeding 8 points. Classification and scoring were performed in consensus by two fellowship-trained orthopedic trauma surgeons.

In this study, the FFP classification was used to describe fracture morphology, as it is the most widely accepted system for characterizing pelvic fragility fractures. However, treatment indication was supported by the OF-Pelvis score, which integrates not only fracture morphology but also patient-related parameters such as pain severity, mobility, comorbidities, and frailty. Since all patients in our cohort had FFP type 4B fractures, the morphological instability was uniform. The OF-Pelvis score was therefore applied to ensure that surgical indication reflected both mechanical instability and the clinical context of each individual patient.

### 2.3. Surgical Technique

All procedures were performed in a hybrid operating theater using computer-assisted navigation (Brainlab, Munich, Germany) in combination with intraoperative CT imaging (AIRO, Stryker, Kalamazoo, MI, USA). Patients were positioned supine on a radiolucent operating table and underwent 360° sterile draping to allow unrestricted intraoperative imaging and surgical access. After sterile preparation, an intraoperative CT scan was acquired and automatically registered to the navigation system, providing real-time 3D anatomical data for trajectory planning.

The optimal implant pathway was planned on the navigation workstation to safely traverse the sacrum while avoiding the sacral foramina and engaging the densest available cortical bone, particularly along the anterior sacral border.

Through small bilateral skin incisions (approximately 2–3 cm) over the posterior superior iliac spine, navigated guidewires were advanced percutaneously across the sacrum under continuous navigational guidance. A solid trans-sacral bar (7.5 mm diameter, titanium or stainless steel) was inserted from ilium to contralateral ilium with confirmed bicortical purchase. The bar was secured on the contralateral side with a counter washer screw, allowing controlled compression across the fracture and preventing distraction. In all cases, supplementary short sacroiliac screws were placed to enhance rotational stability.

Constructs used:

1. Hybrid construct (TSB + bilateral iliosacral screws): A cannulated TSB (7.5 mm) was inserted percutaneously across the sacral body (typically S1) under navigated control. Two partially threaded iliosacral screws were then inserted bilaterally, usually at S1 but occasionally at S2 if anatomy dictated.
2. Dual-bar construct (two parallel TSBs): In cases with adequate screw corridors or poor bone purchase, two parallel bars were introduced (S1 and S2). This configuration increased torsional resistance while maintaining a minimally invasive profile.

### 2.4. Data Collection and Variables

Demographic variables (age, sex), mechanism of injury (e.g., fall from standing height), and comorbidities were recorded. Fracture morphology was assessed for the presence of H-type configuration and comminution.

Preoperative imaging included high-resolution CT for fracture delineation and MRI when sacral edema or occult fractures were suspected. Operative data encompassed surgical duration and intraoperative complications. Postoperative variables included hospital length of stay and discharge mobility status (walker-assisted, crutch-assisted, or bedridden).

### 2.5. Bone Quality Assessment

Routine DXA scanning was not available for all patients. Therefore, bone quality was assessed opportunistically using preoperative CT scans:

- Circular regions of interest (ROIs) were placed in the L5 vertebral body trabecular bone, avoiding cortical margins and venous channels.
- If L5 was fractured or unsuitable, the nearest intact lumbar vertebra was chosen.

Thresholds were interpreted according to validated literature: ≤110 HU was considered highly specific for osteoporosis, while values of 160–170 HU were considered sensitive for low bone density.

### 2.6. Postoperative Care and Rehabilitation

All patients were mobilized within 24–48 h postoperatively under physiotherapy supervision. Partial weight-bearing with a walker or crutches was advised for 4–6 weeks, progressing to full weight-bearing as tolerated. Standard thromboprophylaxis was administered until adequate mobility was regained. All patients received low-molecular-weight heparin for at least 6 weeks. Vitamin D supplementation was prescribed when indicated, and initiation of antiresorptive or anabolic therapy was recommended.

### 2.7. Follow-Up Protocol and Radiological Assessment

Follow-up occurred at 3 and 12 months postoperatively, including clinical evaluation, pain assessment (0–10 numeric rating scale), and radiographic evaluation. Stability was defined as absence of implant migration, loosening, or new fracture displacement. CT scans were performed selectively if pain recurred or instability was suspected.

### 2.8. Outcome Measures

Primary outcomes:

- Pain assessed on a numeric rating scale (NRS 0–10).
- Mobility, compared with pre-injury baseline (independent ambulation, walking aid, wheelchair, bedridden). Return to baseline was defined as functional recovery.
- Radiographic healing (union, absence of displacement, absence of implant failure).

Secondary outcomes:

- Complications, recorded according to Clavien–Dindo classification:
  o Intraoperative: bleeding, malposition, neurovascular injury.
  o Early postoperative (≤30 days): wound infection, hematoma, thromboembolism, pneumonia, urinary tract infection, delirium, new neurologic deficit.
  o Late postoperative (>30 days): implant loosening, migration, breakage, reoperation, new fractures, mortality.
- Length of stay (LOS).
- Survival: all-cause mortality at 30 days and 12 months.

### 2.9. Statistical Analysis

Descriptive statistics were performed using IBM SPSS Statistics version 29 (IBM Corp., Armonk, NY, USA). Continuous variables were expressed as means ± standard deviation (SD), while categorical data were presented as frequencies and percentages. Due to the exploratory nature of the study, no inferential statistical comparisons were made.

## 3. Results

A retrospective analysis was conducted on 31 patients treated for FFP type 4B sacral fractures at our institution between 2015 and 2022. This cohort comprised comprehensive clinical, demographic, radiographic, operative, and early follow-up data. Data completeness was high, with minor gaps in follow-up variables such as pain scores and implant loosening status.

### 3.1. Patient Demographics and Baseline Characteristics

The patient cohort was predominantly female (*n* = 27; 87.1%), reflecting the gender distribution commonly associated with osteoporotic pelvic fractures in older populations. The mean age at the time of treatment was 77.9 years (SD: 6.2 years; range: 66–89), consistent with a geriatric population vulnerable to fragility fractures. Traumatic injury was identified as the precipitating event in 17 patients (54.8%), while the remaining 14 patients (45.2%) sustained fractures in the absence of identifiable trauma, suggestive of insufficiency mechanisms. The results are summarized in Table 1.

### 3.2. Fracture Classification and Radiographic Findings

Fractures were classified using the Fragility Fracture of the Pelvis (FFP) system. By study design, all patients presented with type 4B (“H-shaped”) sacral fractures, reflecting the most unstable injury pattern.

Posterior pelvic ring fractures were bilateral in all patients (100%). Concomitant anterior pelvic ring fractures were present in 15 patients (48.4%), either unilateral or bilateral.

A comminuted fracture zone, which may increase surgical complexity and compromise healing potential, was documented in 13 patients (41.9%). We used the OF-Pelvis classification as a supportive tool for therapy decision. In our cohort, all cases corresponded to OF type 4. Since this study includes only surgically treated patients, all cases had a score >8, thus meeting the criteria for operative management.

### 3.3. Surgical Management and Hospital Course

All 31 patients underwent surgical treatment, with operative techniques and implants tailored to fracture morphology. The most common approach was a hybrid construct combining one trans-sacral bar with bilateral iliosacral screws (*n* = 25; 80.6%), while a smaller subset was treated with dual trans-sacral bars (*n* = 6; 19.4%).

The mean operative time was approximately 71 min, with a range of 41 to 110 min. The average postoperative hospitalization was 9.1 days (range: 5–14 days), aligning with expectations for elderly surgical patients undergoing pelvic stabilization.

### 3.4. Intraoperative and Postoperative Complications

No intraoperative complications occurred. Specifically, there were no neurovascular injuries, no malposition of implants, and no excessive bleeding.

Postoperatively, there were no wound infections, hematomas, thromboembolic events, pneumonias, urinary tract infections, delirium, or new neurological deficits. Importantly, no implant-related complications were detected during follow-up, including loosening, migration, or breakage.

No revision surgeries were required in the entire FFP 4B cohort. This contrasts with previously reported revision rates after spinopelvic fixation in fragile bone, which exceed 10% in some series.

According to Clavien–Dindo grading, no complications of grade I or higher were observed.

### 3.5. Opportunistic CT Bone Density (HU)

Opportunistic CT-derived Hounsfield Unit (HU) values were available for all 31 surgically treated patients. The mean HU was 75.8 ± 30.1 (range 10–141), reflecting markedly reduced bone density consistent with osteoporosis.

### 3.6. Postoperative Mobility and Rehabilitation

Mobility status at discharge was recorded for all patients. The majority were discharged using a rollator walker (*n* = 19; 61.3%), indicative of moderate functional recovery. Other assistive devices included crutches in 6 patients (19.4%) and a high walker in 4 patients (12.9%). One patient (3.2%) was discharged bedridden.

### 3.7. Follow-Up Pain and Implant Integrity

Postoperative pain was assessed at both 3-month and 12-month intervals. At 3 months, pain scores were available for 28 patients, with a mean value of 1.9 on a 10-point numeric rating scale, suggesting generally favorable early outcomes. By 12 months, pain data were available for 23 patients. A substantial proportion (*n* = 14; 60.9%) reported complete pain resolution (score = 0), and only a minority (*n* = 2) reported persistent moderate pain levels (score ≥ 5). The results are summarized in Table 2.

**Figure 1:**
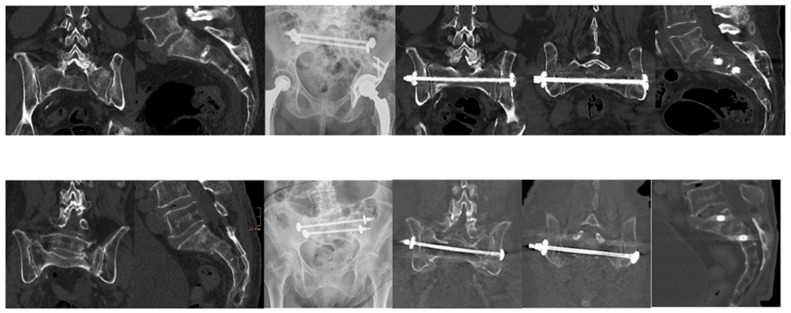
Two Clinical Examples: CT images on the left illustrate a displaced pelvic fracture with a comminuted zone in the lateral mass and an anterior angulation in the sagittal plane. The accompanying X-ray shows fixation using two transsacral bars (TSB). Follow-up CT images on the right, taken six months postoperatively, demonstrate satisfactory fracture consolidation.

Importantly, radiographic assessment for implant loosening revealed no cases of loosening among the 31 patients with evaluable follow-up data, indicating high biomechanical integrity of the fixation constructs employed. Figure 1 shows two representative examples of H-shaped fractures with good healing demonstrated on CT after six months.

## 4. Discussion

### 4.1. Study Aim and Key Findings

This study evaluated elderly patients with osteoporotic H-shaped sacral fragility fractures (FFP type 4B), treated with navigation-guided trans-sacral bar (TSB)-based fixation. The main findings were: (1) stable fixation without implant failures or revisions; (2) significant pain reduction and restoration of mobility; (3) consistent radiographic healing despite severe osteoporosis; and (4) a favorable perioperative safety profile with no complications. These results demonstrate that TSB-based fixation is a safe and effective treatment strategy for this highly unstable fracture type.

### 4.2. Comparison with Literature

Previous literature has emphasized spinopelvic fixation (SPF) as favorable option for unstable sacral fractures [23,24,26,29]. However, SPF carries substantial morbidity in elderly patients, including increased blood loss, wound complications, and prolonged immobility [25,26,29]. Our findings support recent reports that TSB fixation may provide sufficient stability for fragility fractures with fewer complications.

Wagner et al. reported a one-year mortality of 9.6% and a 15% revision rate after TSB fixation [9]. Mehling et al. demonstrated bony union and satisfactory stability with trans-sacral bar fixation in dorsal pelvic fatigue fractures [22]. Mendel et al. showed that bisegmental trans-sacral screw fixation achieved outcomes comparable to SPF in bilateral sacral fragility fractures but with fewer complications [30], a conclusion also supported by Andresen et al. [13]. In our cohort, operative time averaged 71 min, hospital stay was 9.1 days, and no revision surgeries were necessary. These results are favorable compared to existing reports, particularly regarding implant survival.

### 4.3. Fixation Strategies: Hybrid Versus Dual-Bar Constructs

Biomechanical studies suggest that a single trans-sacral implant may not provide sufficient torsional stability, while hybrid (TSB + iliosacral screws) or dual-bar constructs achieve greater load sharing and resistance to rotational forces [25,31]. In our series, most patients (80.6%) received hybrid fixation, while a smaller subgroup (19.4%) received dual-bar fixation. Both approaches resulted in stable fixation without mechanical failure. Although subgroup comparisons were limited by sample size, our results support the concept that two-point fixation is preferable to a single-bar technique in osteoporotic FFP type 4B fractures.

### 4.4. Bone Quality and Osteoporosis Considerations

Osteoporosis was confirmed in all patients, with mean opportunistic CT-derived Hounsfield units (HU) of 75.8 ± 30.1. This value lies well below the accepted thresholds for osteoporosis (≤110 HU specific, 160–170 HU sensitive) [20]. These findings underscore the fragility of the sacrum in this patient population. While DXA and biochemical markers were unavailable, CT-based HU provided a consistent and practical surrogate for bone density assessment. Importantly, fracture morphology in our series was uniform (FFP type 4B), suggesting that factors beyond absolute HU values—such as sacral anatomy and loading mechanisms—contribute to this characteristic fracture pattern [18,19,20].

### 4.5. Safety Profile and Complications

A key finding of this study was the absence of intra- or postoperative complications. No cases of implant malposition, neurovascular injury, wound infection, or systemic complications were observed. Equally important, no implant loosening, migration, or revision surgery occurred during follow-up. This contrasts with revision rates > 10% reported for spinopelvic constructs in osteoporotic bone [23,24,29,31]. These data highlight the favorable safety profile of TSB fixation, particularly when performed with computer navigation.

### 4.6. Clinical and Economic Implications

The combination of reliable fixation, short operative time, and absence of revisions suggests that TSB fixation may be more resource-efficient than SPF. Although no formal cost analysis was performed, reduced perioperative morbidity and simplified aftercare may translate into economic advantages, particularly relevant given the rising incidence of pelvic fragility fractures in aging societies [1,2,3,16]. Clinically, TSB fixation offers a less invasive option that still achieves stability, enabling early mobilization and functional recovery in frail elderly patients.

### 4.7. Limitations

Several limitations must be acknowledged. This was a retrospective, single-center study with a relatively small cohort. Functional outcomes were not evaluated with standardized instruments such as EQ-5D or Oswestry Disability Index. Follow-up was limited to 12 months, and survival analysis relied on hospital records. In addition, subgroup analysis comparing hybrid and dual-bar fixation was limited by sample size. Finally, no SPF control group was included, preventing direct comparison between fixation strategies.

### 4.8. Future Directions

Future studies should include prospective multicenter trials comparing TSB-based fixation with SPF specifically in FFP type 4B fractures. Longer-term follow-up is needed to assess implant survival and late complications. Biomechanical investigations into hybrid, dual-bar, and cement-augmented constructs may refine fixation strategies [25,31]. Incorporating systematic bone quality assessment with DXA, biochemical markers, and opportunistic CT will improve external validity and support personalized treatment decision-making.

## 5. Conclusions

In elderly patients with osteoporotic H-shaped sacral fragility fractures (FFP type 4B), navigation-guided trans-sacral bar (TSB)-based fixation provided stable osteosynthesis with no implant failures, no revisions, and consistent fracture healing. Pain decreased substantially, mobility improved in most patients, and perioperative morbidity was minimal. Hybrid and dual-bar constructs both achieved reliable stability. These findings indicate that TSB fixation is a safe and effective alternative to spinopelvic fixation for FFP type 4B fractures, though larger prospective multicenter studies are needed to validate these results and further define optimal construct selection.

## Figures and Tables

**Table 1:** Baseline Characteristics of the Study Population (FFP type 4B, *n* = 31).

Parameter	Value
Age, median (range)	77.9 (66–89) years
Female, %	87.1
Male, %	12.9
Trauma mechanism	Fall 54.8%; Atraumatic 45.2%
Hounsfield Units (HU), mean	75.8 ± 30.1
FFP classification	FFP Type 4B (100%)
OF classification	OF Type 4 (100%)
Comminuted zone, %	41.9
Anterior ring involvement, %	48.4

**Table 2:** Operative and Postoperative Outcomes (FFP type 4B, *n* = 31).

Parameter	Value
Operative time, mean (range)	71 min (41–110)
Hospital stay, mean (range)	9.1 days (5–14)
Loosening rate, %	0
Walker at discharge, %	61.3
High walker at discharge, %	12.9
Crutches at discharge, %	19.4
Bedridden, %	3.2
Pain level at 3 months, mean	1.9
Pain level at 12 months, mean	1.1

## Data Availability

The data presented in this study are available on request from the corresponding author. The data are not publicly available due to privacy and ethical restrictions.

## Abbreviations

The following abbreviations are used in this manuscript:

SPF	Spinopelvic Fixation
TSB	Transsacral Bar
SIFs	Osteoporotic Sacral Insufficiency Fractures
FFP	Fragility Fractures of Pelvis
OF	Osteoporotic Fractures
